# What underscored successful malaria elimination in Palestine 100 years ago? Effective Education

**Published:** 2021-06-01

**Authors:** Anton Alexander

**Affiliations:** 1 BC Business Centrum, Elscot House, Arcadia Avenue, London N3 2JU, United Kingdom.

## Abstract

Transmission of malaria by anopheline mosquitoes had been established by 1897, and in 1922, the first start of a successful national malaria elimination campaign began. Until then, only malaria control had been considered anywhere as a feasible project, such malaria control having been conducted primarily through larval source management. From 1922 onwards, in Palestine, by ensuring the breeding sites remained destroyed continuously over years and years, malaria elimination was eventually achieved. However, in order to achieve such continuous destruction, transmission of the disease had to be imaginatively and sensitively explained to all the inhabitants who thereupon willingly accepted the task of ensuring the breeding sites remained destroyed. Without that education, the inhabitants would not have provided the continuous work required, and Palestine would have remained in its severe malarious state.

## Introduction

Since the end of the nineteenth century, malaria has been known as a local problem due to the fact that mosquitoes travel only a limited distance from their breeding sites in order to bite and feed on humans. If that mosquito has previously been infected, when it bites, it also injects the malaria parasite at that moment, thereby transmitting the disease. Therefore, destruction of mosquito breeding sites can prevent or control malaria in that locality. In 1922, Palestine became the place for the first start of a successful national malaria elimination campaign, a feat that was achieved through continuous and sustained larval source management, to ensure that those breeding sites remained unproductive for mosquitoes for years on end. The World Health Organization (WHO) Handbook on Integrated Vector Management (IVM) includes a brief historical note about malaria elimination. It merely states:

"*Before the Second World War, vector control was conducted predominantly by environmental control of the proliferation of mosquitoes.* [e.g., the 1922 Palestine method] *.... There is evidence that environmental management had a clear impact on disease; however, elimination of disease was never on the agenda. The advent of DDT and other organochlorine pesticides during the 1940s changed this situation. ... The focus of vector control on insecticides meant that environmental management and other alternative methods were underexploited **or even forgotten**.*" [1]

But what actually was forgotten? Malaria control has been conducted for many years, but the world still wrestles with the next step, namely the problem of converting malaria control into malaria elimination, particularly in resource-poor malaria-endemic settings where transmission is intense and perennial. Nevertheless, in theory, if malaria can be controlled for long enough, malaria elimination should follow. There has been many a time that inhabitants relaxed their vigilance in the mistaken belief the disease had been beaten. The breeding sites were then abandoned, and as a result, the disease returned with a vengeance [2]. To manage breeding sites sustainably over longer periods, an understanding of the causes of the disease would assist in acquiring the necessary patience to deal with such maintenance. Effective education, therefore, is essential.

The WHO handbook above refers to something having been forgotten, and this paper deals with that forgotten topic. Since 1897, it has been known that effective management of mosquito breeding sites will control malaria. But ensuring that breeding sites remain non-productive for mosquitoes required a population that understood and appreciated why the breeding site should remain attended to for years and years. That understanding and appreciation necessitated education – and effective education is the topic that appears to have been forgotten. Here, I examine the effective education that took place in Palestine in 1922. The Palestine method was successful, and the reader is invited to make the comparison between the reader’s personal experience (if any) of education associated with IVM today and the ‘forgotten’ education of the Palestine experience all those years ago. Perhaps then it will be understood why attempts today at malaria elimination have not been more successful.

### The Malaria Lesson from Palestine 100 years ago

In 1919, Dr. Manson-Bahr, a future director of the London School of Hygiene and Tropical Medicine, described Palestine as one of the most highly malarious countries in the world [3]. He knew the conditions in Palestine as in 1918, in the final months of WW1, whilst an officer in the Royal Army Medical Corps with the British Army in Palestine, he had witnessed a force of 40,500 men lose 20,427 men in 9 weeks due to malaria. Of the 100,000 Turkish prisoners-of-war taken after their defeat in 1918 by the British Army in Palestine, 20 per cent had to be hospitalised immediately, suffering from malaria. After the defeat of the Turkish Army, Palestine was administered by the British Mandate, in effect a colony-like structure.

It is usually not appreciated that if malaria had not been eliminated in Palestine, it is doubtful that Israel could ever have come into existence. In 1922, almost 100 years ago, the British Mandate conducted a Census in Palestine disclosing a total population of 757,182, including all the military. Palestine was then either thinly populated or uninhabitable in many areas. The area underwent a huge transformation, with the population in 2020 for the same area increasing to approximately 13,5 million – an increase of almost 18 times the 1922 level.

Dr. Kligler, an idealistic Jewish Zionist and a brilliant public health scientist, arrived to settle in Palestine in 1920. He commented that:

“*…unless something was done to check the ravages of malaria, the reconstruction of Palestine would be a costly if not altogether an impossible effort.*” [4]

Accordingly, in 1921/22, under Kligler’s direction, Palestine became the first place anywhere in the world to embark on a successful national malaria elimination campaign. Kligler’s method relied basically upon the removal of mosquito breeding sites, and was conducted without reliance on drugs, insecticidal sprays for indoor use (DDT had not been (re)discovered yet) or bednets:

“*The control methods used by Kligler were based on sound principles (e.g. draining swamps, clearing overgrown canals, or diverting springs and wadis), and while not novel in practice, perhaps the degree of implementation and of attention to detail of such methods was unique in a number of ways. … But perhaps Kligler’s most unique contribution to malaria eradication was his emphasis on education, and it is this contribution that seems generally to be either unappreciated or not understood, even today. Unless the malaria control work could be maintained and sustained, there was little point in undertaking it in the first place.*” [5]

Kligler would always maintain that education was as important as the anti-malaria work itself, and it is the intention here to demonstrate the strength and commitment that education provided to local inhabitants, enabling them to become an integral part of successful malaria elimination. Education was intended to overcome two hurdles. Firstly, and perhaps the greatest hurdle, was fatalism, a sense of inevitability about the disease, as malaria elimination had never been achieved anywhere previously. Overcoming fatalism had no straightforward solution, but probably one of the most effective approaches had included encouragement to hear or see the benefits in neighbouring villages or settlements where malaria elimination work was already underway. Secondly, the population had to understand the necessity for maintenance of the anti-malaria works which had destroyed mosquito breeding sites. If the mosquitoes were permitted to return, so would malaria:

“*There didn’t seem to be a single uniform way to [educate] the population other than on a one-to-one basis. Each person had to understand what was the problem; namely, why the works were necessary and why the maintenance was essential. With such a mix of backgrounds and levels of education (or sometimes lack of it), Kligler had to deal with each person individually, at that person’s own pace. Therefore, in order to ensure that everyone appreciated the problem and thereby hopefully secure the co-operation of the population, Kligler and his team proceeded to engage with the inhabitants [Arabs and Jews] as much as possible on a personal, individual basis, rather than rely on intermediary representatives from each different community.*” [5]

For the anti-malaria work to be effective, all maintenance had to be thorough, continuous and systematic over many years, and this could only be achieved if locals understood and appreciated why they were performing the tasks. It was also absolutely essential that *all* inhabitants were treated with dignity and respect. The maintenance work was often tedious, but due to Kligler’s effective education, for years the inhabitants provided their voluntary co-operation in the discharge of their role in the elimination effort.

In 1933, the Egyptian Medical Association decided to hold its 6^th^ Annual Congress in Jerusalem. A number of Kligler’s team were requested to provide papers on their malaria work for the event, and the following by Dr. Abraham Levy gave an insight into practical aspects relating to Education:

“*… institute a systematic series of educational demonstrations to be presented by the sanitary inspectors when he makes his rounds. Instead of coming merely to inspect, to find fault or to look for trouble, he should spend several days or even a week among the inhabitants, to teach them by actual demonstrations the havocs that careless habits of needless spilling of water might bring to the district.*

…

*It depends upon the ingenuity of the [sanitary] inspector to take his audience into his confidence and make them feel that he is only discussing the problem with them, rather than forcing his ideas upon them. This attitude will be more effective to carry out his rules and regulations than dozens of laws and threats.*

…

*… The task is not easy. It may mean months of hard labour and many discouraging moments, but through perseverance and constant attempts, the results will come, and when they do, they will be of a permanent character.*” [6]

The following specific example may or may not have been used by Kligler, but it serves the purpose of visually demonstrating an aspect of the education that Kligler appreciated and stressed. As already mentioned, maintenance of the works that kept potential breeding sites non-productive for mosquitoes was vital. The maintenance had to be planned so that all work was carried out thoroughly, continuously and systematically. Kligler would have warned the inhabitants against taking on too much, in order to allow sufficient time for a task to be carried out thoroughly. Such maintenance may be likened to an entertainer who balances spinning plates on poles/ sticks. The entertainer begins with one pole/stick and plate and then adds more and more. At some stage, the entertainer has to return to the first plate, which is slowing, in order to revive it, and he must repeat that up the line of slowing plates. The entertainer realises he should not extend his line of spinning plates indefinitely, otherwise he can’t maintain his performance. These spinning plates may be likened to mosquito breeding sites. Taking on more work than is comfortable in the maintenance of destroyed breeding sites prevents the work being conducted thoroughly and could allow small gaps for the mosquito to proliferate. The maintenance work must be comfortably and realistically manageable. Keeping the plates spinning during a performance and keeping breeding sites destroyed over years and years is a continuous and wearing task. If fatigued or worn out, one relaxes and the plates tumble and the mosquitoes return. To ensure that nationally breeding sites would remain destroyed, the inhabitants in each locality throughout the country would have to assume responsibility for ‘keeping their plates spinning’ in that locality. Until ‘all the plates throughout the entire country remain spinning at approximately the same time’, malaria will never be eliminated.

Kligler also had to contend with a diversity in the population, which he described in his 1930 textbook:

“*Palestine is a country of mixed peoples, many religions, and all gradations of civilisation. There are Jews from the Orient and Occident, and side by side there are city Arabs, the peasants, and the roving Bedouins. There are Moslems, Jews, and Christians, people of every sect and denomination. And there are as well all gradations of culture, from the Nomadic, pastoral Bedouins to the most modern industrialised groups. A more heterogeneous population can hardly be found anywhere in the world.*” [7]

There was no national identity and no cohesion between the inhabitants other than to their own religion, individual group, or tribe. There was no notion of a Palestinian entity or nation. Palestine was made up of mixed peoples who happened to be in Palestine at that moment. Kligler never wrote specifically about practical steps he took with regard to education, and we can only surmise what they might have been. Due to the diversity in the population, there could have been no fixed method, no standard or straightforward way, no ‘silver bullet’ to educate the population, and different groups or individuals would have required different approaches according to different backgrounds or cultures. He presumably engaged with the population in a more sensitive manner than the population would have expected. After making initial contact with a group or individual and discussing matters generally, he would presumably have tried to gauge or assess the best way forward in an attempt to stimulate an interest and without giving offence. It was likely that this was his yardstick when dealing with the inhabitants. Because the education was so successful, it was quite obvious that something very unique must have been afoot. Education was accorded a high priority. It would seem each inhabitant was encouraged to be a participant in the elimination project, and was not expected merely to respond to orders or instructions. Education ensured that inhabitants felt involved and not merely part of the labour force.

Kligler’s approach may be considered as the antithesis of neo-colonialism. Each inhabitant was accorded dignity and respect, he counted, and he became a valued team member upon whom others could depend and whose contribution would determine the failure or success of the elimination project. Each inhabitant was to be shown the principles of malaria control, the intention being that slowly the inhabitants would develop the habit to carry out the necessary work themselves. Kligler wished to inculcate the habit of proper self-care.

In 1925, Dr. F. Russell, director of the Rockefeller International Health Division inspected the anti-malaria work in Palestine, and wrote:

"*I do not know when I have seen better and more successful anti-malaria work than that which is being done in Palestine... The co-operation of the people with the authorities leaves nothing to be desired... It is an ideal way to carry out malaria work because it makes the population served by the anti-malaria measures participants in the project from the beginning; they then have a better understanding of the problem and will be more ready than they otherwise would be to take care of the necessary maintenance.*" [8]

Also, in 1925, the Malaria Commission of the League of Nations (the equivalent of today's WHO) came to inspect the works. At the end of its two-week tour of inspection, Professor Nocht (Hamburg Tropical Diseases Institute), President of the Commission stated:

“*Palestine showed the fruits of an energetic and victorious campaign which would stimulate others to follow the methods there employed… It was not the custom of the Commission to make comparisons but he would on this occasion, say that the interest that Palestine had provided was unsurpassed by that of any of their other visits [to other countries], and never had the Commission met with such preparations as had there been provided.*” [9]

With fatalism in its mind, the Commission praised what it had seen and concluded its report with the observation that the anti-malaria works and methods that they had seen in Palestine:

“… *destroyed pessimism, raised hopes", and those involved in this work were "benefactors not only to the Palestinian population but to the world as a whole.*" [10]

The anti-malaria work continued, and Israel was declared malaria-free by WHO in 1967.

### So why is the above known to only a few?

Despite the above, only two years later, in 1927, the League of Nations issued a Report to the Health Committee by Dr. A. Lutrario on the Work of the Malaria Commission:

"*... the sole purpose of the campaign against malaria, as far as the Commission is concerned, is to reduce the incidence and severity of the disease. Measures designed to accomplish more than that (**particularly measures aiming at eradication**) are not a wise proposition ... The Commission had occasion to enter into relations with a number of states which ... had undertaken an energetic campaign against malaria. The disease yielded to the measures which were taken, but reappeared with added virulence as soon as these measures were somewhat relaxed. This is a very serious result which brings in its train disillusion and discouragement.*" [11]

Kligler’s antimalarial method was new and untested. Whilst not mentioning Kligler by name, Dr. Lutrario appeared pessimistic about the Palestine inhabitants continuing with maintenance of the works to ensure the breeding sites remained nonproductive or destroyed. This 1927 Report appears to be at variance with the comments and observations made by the experts in 1925, and it is necessary to consider the background to that 1927 report.

A Century ago, Rome was then a centre for malaria research. Italy had a huge malaria problem and a great deal of southern Italy was deemed uninhabitable. The author of the 1927 report, Dr. Lutrario, was one of the original six members in the creation of the League of Nations Malaria Commission. He was the powerful Italian Director of Health, and his presence reflected the importance granted to Italy on the Malaria Commission.

Towards the end of the 1920s, two schools of thought had developed on the preferred method for dealing with malaria. The Italian (or European) school favoured giving priority to health care services including treatment of those suffering from the disease, whilst the American school gave priority to control of the mosquito. Lutrario and the Malaria Commission were of the Italian/ European school, whilst Kligler (formerly a star of the Rockefeller Foundation before arriving in Palestine) was of the American school. These two schools frequently confronted one another in the 1920s and ‘30s.

It is important to point out that, although Lutrario was a member of the Malaria Commission, he did not travel with the Commission in 1925 to inspect the works in Palestine. His impressions formed of the works and the attitudes of the inhabitants were therefore based on second-hand information, either written or verbal from the other Commission members. As will be noted from the comments of Dr. Russell of the Rockefeller Foundation, the education of the inhabitants was unique, and Lutrario would have been unlikely to have ever seen or experienced anything similar.

Further, before writing his 1927 Report, Lutrario should have made himself more familiar with the conclusions of the Malaria Commission when it inspected Palestine in 1925. The following, from page 37 of the 1925 Commission report, should have drawn his attention that something of significance was taking place in Palestine, particularly where it was stated that ‘successful’ works previously shown to the Malaria Commission in other countries were not, in effect, up to the standard required in Palestine:

“… the authorities had succeeded in interesting the local population (sometimes semi-nomadic Bedouin tribes!)^*^ in anti-malarial work and induced them to carry out irrigation and drainage schemes, which, although by no means complete, might be compared favourably with several important works [in other countries] shown to us last year as successful anti-malarial operations. Here, on the contrary, the authorities, in some instances, refused to consider them as such but simply as a means to educate the population to a more complete insight, which would enable them to give in future their wholehearted co-operation as soon as this should be wanted.” [10]

^*^ The exclamation mark above was inserted by the Malaria Commission, apparently surprised that Kligler sought fit to include the Bedouin.

Lutrario seems to have suggested Kligler’s approach to be a little pointless as it could not guarantee a long-term commitment by the Palestine inhabitants to maintain the work. This was also at a time when Mussolini’s fascist party governed Italy, and antimalarial campaigning formed a central part of fascist domestic policy, so the praise received by Kligler may have represented unwanted competition for Mussolini’s future projects. It would also seem there was a whiff of racism or neo-colonialism, that the Arab-Jew cooperation would unravel over a prolonged period. But whatever the reason, the co-operation was strong and Kligler’s pro-gramme did continue. Kligler’s education ensured an understanding in each inhabitant of why maintenance of the anti-malaria works was necessary, with each inhabitant assuming a responsibility for maintenance, and usually providing voluntary assistance when requested.

Kligler’s antimalarial work had commenced only five years before the 1927 report. At that moment, it was pointless trying to disprove Lutrario’s claim of future failure for Kligler. With hindsight, we now know Kligler’s method was effective, but in 1927, that could not yet be demonstrated. Because of Italy’s and Lutrario’s standing and influence within the international malaria community, in view of the 1927 report, countries accordingly would in all likelihood have ‘downgraded’ the chances of success for Kligler, expecting the whole Palestine project to fail. But it didn’t fail, Kligler’s work quietly continued and Palestine/ Israel was declared malaria-free in 1967. However, Kligler’s method ceased to be noticed and he thereafter became a forgotten man by the international malaria community.

One can only wonder what motivated Lutrario to publish his 1927 report. Was it intended to be just another confrontation with the American school, or did Lutrario genuinely consider the maintenance would not be sustained? We will never know. But it certainly reduced the attention that Kligler received in many parts of the world.

### Prompt & energetic co-operation – the remarkable feature of the antimalarial campaign

If ever proof was needed of a population’s commitment to be free of malaria, then the co-operation that existed in Palestine is that proof. In 1941, the British Mandate’s Department of Health had reviewed the malaria position in Palestine, and praised the co-operation:

“*In the true rural areas the lasting effects of swamp and marsh reclamation, the proper irrigation of gardens and similar areas, and the periodical canalisation, filling and clearing of stream beds, have so impressed the people by the resulting improvement in health, and the land thus made available to farmer and shepherd, that their prompt and energetic co-operation is one of the most remarkable features of the antimalarial campaign in this country.*” [12]

Lutrario was not the only hindrance to Kligler’s efforts of eliminating malaria. The following is a glimpse of some of the other difficulties with which the co-operation had to contend, and which demonstrated the strength and resilience of the cooperation, enabling elimination to still take place. Even when confronted by social unrest, and the intimidation and threatened violence against the population (both Arab and Jew), the co-operation continued. Kligler had to accept the situation as he found it, and in his quest to educate and secure cooperation, he knew from the outset that he had to deal with elements in the population who potentially were very hostile.

Kligler demonstrated anti-malarial works near or at the breeding sites, thereby interesting and involving the inhabitants in necessary maintenance, to ensure breeding sites remained destroyed. When offering explanations, it would be done at whatever level and pace seemed appropriate, and such explanations would be offered, even individually, if so requested by an inhabitant. Further, Kligler or his team would return if necessary where inhabitants had failed to understand any aspects of the anti-malaria works and malaria-elimination generally. It was also taught that malaria-elimination had to be kept alive so that even when the disease appeared beaten, it was still essential for the population to maintain the works.

In April 1920, there had been a massacre of Orthodox Jews by Arabs in Jerusalem. Kligler arrived in December of that year to settle in Palestine. Possibly the extreme violence that Kligler found even before he began his work helped to ensure he pitched the need for education even higher, even on a one-to-one basis. He knew that unless he convinced everyone of the need to co-operate, his goal of sustained malaria-elimination would have been in tatters. Notwithstanding this, and against this backdrop, Kligler, a Jew, still sought to enlist the whole population, both Arab and Jew, in his attempt to obtain its co-operation in defeating malaria. Kligler realised that without such co-operation, the disease would never be eliminated. Despite the extreme violence that already had taken place in 1920, in late 1921, Kligler began in earnest his anti-malaria campaign, including its emphasis on education of the population and the resulting co-operation.

The period between 1922 and 1929, 8 years, were years of peace. By 1926, the British Government felt able to reduce the forces available for maintaining order to a very low strength because, according to the 1925 report of League of Nations Malaria Commission (page 22):

“*For some time past, Palestine has been the most peaceful country of any in the Middle East*”!! [10]

In 1928, the British Health Department reported:

“*We rely almost entirely on voluntary labour by the villagers and colonists …, it is seldom that we are disappointed in obtaining voluntarily all the labour required for the annual clearance of streams, wadis etc. and for new minor works in connection with swamps.*” [13]

But in 1929, the peaceful times ended - yet the cooperation continued!

**Figure 1:**
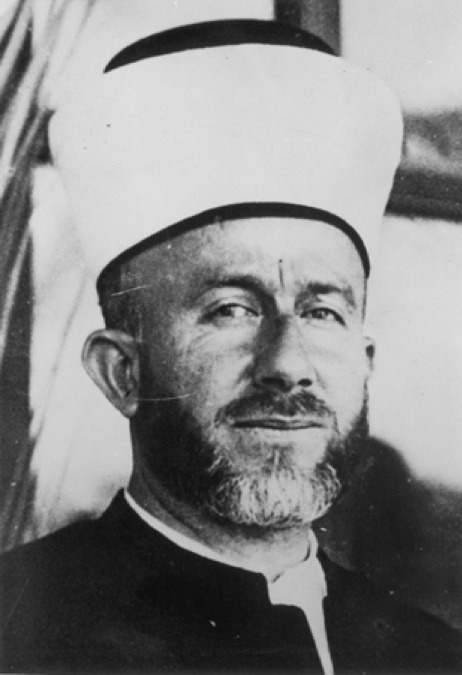
Haj Amin el Husseini, Grand Mufti of Jerusalem.

In 1922, Haj Amin el Husseini, a member of an influential Arab family in Palestine, had been appointed the Grand Mufti of Jerusalem, becoming the leader of the Palestine Arabs (Figure 1).

He had been implicated as a leader of the 1920 Jerusalem massacre and was sentenced to ten years imprisonment for incitement but was pardoned by the British. He hated the Jews and was to become a hindrance to the elimination of malaria, fomenting violence and terror. He was later to be appointed leader of the Muslim Brotherhood in Palestine. In August 1929, a massacre of Jews by Arabs took place in a number of towns in Palestine. The subsequent Government Commission enquiring into these 1929 disturbances implicated the Grand Mufti, saying:

“*… the Mufti was influenced by the … desire to annoy the Jews … and the Mufti … must accept a share in the responsibility for the disturbances.*” [14]

Notwithstanding the unrest, the co-operation continued.

The Department of Health Annual Report of 1929 stated:

“*Following a very wet winter, there was expectation of a high incidence of malaria, … It was possible by timely measures, however, to carry out extensive clearance of water courses and marsh areas, ... It was gratifying to find that the villagers **throughout the country** fully cooperated in these measures, the benefit of which they now understand, and the result was that the Department had only to provide foremen, while the people did the rest.*

*… Our experience during 1929 would appear to support the principle that we have adopted during the last few years in Palestine, namely that the Dept. supplies supervision and larvicide in rural antimalaria work, and requires the population to supply the labour to protect themselves and their villages from malaria.*” [15]

There followed further violence during the 1930s, which began to be directed not only against the Jews but also against the British authorities. Even staff engaged in anti-malaria work became a target for Arab violence.

Yet the cooperation continued.

A Royal Commission was arranged to enquire into disturbances which broke out in April 1936, and it wrote of sinister influences on the Mufti coming from Mussolini’s fascist Italy and Hitler’s Nazi Germany. The 1937 Report of the Palestine Royal Commission [16] commented:

“*… the manifestoes issued by the Higher Arab Committee under (the Mufti’s) Chairmanship, … were clearly prejudicial to law and order. Nor … did the Higher Arab Committee at any time condemn the acts of sabotage and terrorism which became more frequent … , and the Mufti as Chairman must … bear his full share of responsibility for those disorders.*” (Page 179)

“*We have seen how the [Palestine Arab] ‘Youth Movement’ … take a sympathetic interest in Fascism.*” (Page 134)

“*… intimidation at the point of a revolver has become a not infrequent feature of Arab politics.*” (Page 135)

The 1937 report included (page 131) ominous evidence given by the Mufti to the Commission:

“*[If the Arabs are] given independence, as the Mufti of Jerusalem said, the Arabs will deal with the Jews themselves*”.

The 1937 Report also included (page 141) further evidence given by the Mufti to the Commission, together with the alarmed reaction of the interviewer who felt he should draw a reader’s attention specifically to the Mufti’s response below:

“… the following questions (were) put to the Mufti of Jerusalem and his replies should be noted^*^:

*Q. Does His Eminence think that this country can assimilate and digest the 400,000 Jews now in the country?*

*A. No.*

*Q. Some of them would have to be removed by a process kindly or painful as the case may be?*

*A. We must leave all this to the future.*”

^*^ The words “and his replies should be noted” above were added by the Royal Commission itself in 1937 as if by a premonition of what unbelievably would befall Europe under the Nazis two years later.

Yet the co-operation continued!

The Government Health Department Annual Reports for 1936, 1937, 1938 and 1939 confirmed the difficulties and dangers experienced by the anti-malaria staff with their regular supervision of mosquito breeding places during the disturbances, and these reports also referred to the necessity for provision of police guards for protection of the antimalarial labour workers.

And notwithstanding the sabotage and violence, each year, **Kligler’s anti-malaria co-operation continued**, as noted in these annual reports.

**Figure 2:**
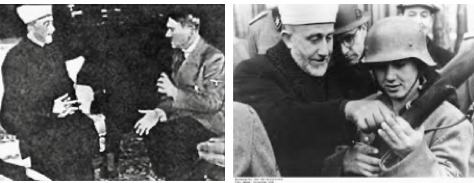
During World War 2. Left: The Mufti with Hitler in Berlin; Right: Assisting German soldier.

The Mufti was obsessed with his hatred of Jews. It is unknown if this alone caused him to encourage disruption of the anti-malaria work, which work he knew was for the common good, but which he was still prepared to hamper. The population (Arab and Jew), however, realised the work was for its benefit, and notwithstanding the conduct of the Mufti, the population continued to co-operate with the anti-malaria work. The hatred of the Grand Mufti towards the British, and his incitement to violence were such that the authorities attempted to arrest him, he fled and eventually spent the duration of WW2 in Nazi Germany assisting the Nazi war effort (Figure 2).

After the Mufti had fled, there were attacks by Arabs who supported the Mufti on those Arabs who didn’t, causing the latter to flee the country in fear for their lives. The numbers fleeing were so great that it caused the total of the Palestine population (Arabs and Jews) in 1938 to drop below the total of the Palestine population in the previous year, in 1937. Such reduction in the population took place even though there were Jews entering Palestine who had managed to escape persecution in Nazi Germany. The intensity of the violence of Arab against Arab had therefore, in 1938, caused such numbers of Arab refugees to flee the Mufti’s supporters that they in fact exceeded the number of fleeing Jews arriving in Palestine. A terrible climate of terror and fear must have existed for any Arab working with Jews or the British Mandate.

Yet still the co-operation continued!

The reader can only try to imagine the terror and violence meted out by the Mufti against his fellow Arabs all those years ago. This may assist to appreciate Kligler’s enormous achievement in countering the effect of the Mufti’s terror and violence. Through Kligler’s education, the inhabitants realised malaria could eventually be consigned to history, and the commitment of the Arab population to be free of malaria translated into something stronger than the weapon of terror and violence wielded by the Mufti. So it is quite understandable why the British Mandate stated in its 1941 Palestine Department of Health Report:

“… [the inhabitants’] *prompt and energetic co-operation is one of the most remarkable features of the antimalarial campaign in this country.*” [12]

Sadly, the Mufti’s violence and indifference to the harmful consequence of his conduct on the Arab inhabitants has travelled down time. To assist the reader in appreciating the level of violence which Kligler’s co-operation had to withstand, the reader should try to examine the organisation, Hamas, which rules Gaza today, and its reported terror and violence which appears to be replicated from the Mufti’s own violence and terror. This is unsurprising as Hamas (classified as a violent terrorist organisation by many countries) is a branch of the same anti-Semitic Muslim Brotherhood that was headed by the Mufti. Yet Kligler managed to create co-operation, Jews and Arabs, from such unlikely raw material; evidence of the strength and effectiveness of Kligler’s education.

### Education and IVM today

IVM literature usually mentions education as an element in its programme, but it is generally not the education that Kligler would have recognised as such. This paper does not set out to provide a practical step-by-step guide enabling anyone within the malaria community to emulate the educational standard achieved by Kligler. It is unknown what a practical guide by Kligler would have looked like, but it has been possible to pull together snippets, which provide clues to the steps he took. This paper does, however, set out to demonstrate that there existed a higher standard of education than is generally treated as acceptable for IVM today.

It seems today that no matter what scientific breakthroughs with insecticides are presented to the malaria community, those involved with malaria elimination always seem to express frustration or disappointment with the inhabitants intended to be benefitted because of some failure (e.g. misuse of mosquito bednets) to co-operate with a particular method. However, the reader should not feel too despondent about this because such disappointment or reaction may sometimes have happened even in Kligler’s day. The 1933 paper by Dr Levy previously referred to opens with:

*“Measures for the control of malaria [in Palestine] are not to be considered complete or efficient unless an adequate and popular health education program is included. … [The inhabitant] who does not assist in exercising the proper means of control of malaria, is not to be blamed or censured, if he is not instructed and made aware of the fact, that marshes and muddy pools of water collected in a yard through careless use and disposal, will produce breeding places for anopheles.*” [6]

But Kligler would have recognised from the conduct of the inhabitants if the education in a location had been inadequate or defective, and steps would have been taken to remedy the position. Kligler was to write:

“*The education of the inhabitants was … by no means the least important element which conditioned the success of the work. Without active co-operation on the part of the people, the work would only have been partially successful. It was possible to obtain their active co-operation only after they understood fully the significance and value of the work.*” [17]

Kligler’s education would have provided the inhabitants with the confidence to carry out work for themselves rather than wait for the authorities to conduct it for them. Education would have convinced the inhabitants that knowledge of malaria was not the privilege only of medical doctors. And it is reasonable to assume Kligler’s education would have dramatically increased the uptake and proper use of bednets today.

Often IVM literature refers to ‘Listening to the Community’ or ‘Community Engagement’. Such an activity is to be applauded. The education which this paper is considering, however, involves also ‘Listening to the Individual’, and if necessary, time should be made available for anyone who is struggling to understand an aspect of the project. The reference in the WHO IVM Handbook to education does not appear to have the same priority that it had in Kligler’s day. IVM is a wide and complex topic and any educational alterations to IVM would have to be carried out by those involved. Perhaps WHO should examine its approach to education.

It is unlikely the education suggested by the WHO for its IVM would have been adequate to assist with elimination of malaria in Palestine. There is a danger that education is merely treated as some task that has to be mechanically undertaken, verging on some neo-colonial exercise. Kligler stressed that education was as important as the anti-malaria work itself, and possibly if education is treated with the same priority as it was by Kligler, malaria eradication may be the prize.

An instructive and very useful paper by Dunkel *et al.* [17] with respect to successful malaria elimination in Mali, Africa, was published in 2013 which dealt with (inter alia) the necessary approach to education for malaria elimination. It warned against attempting education using top-down material, even though that material had been acknowledged as a standard within the malaria community. It is one thing to gain the confidence of a group or of individual inhabitants, it is another thing to ensure the material to be explained will be understandable to them.

## Conclusion

Kligler’s standard of education should now be considered an important alternative to the present standard of education employed in many IVM locations, and which appear to be locked into traditional models whilst marking time, awaiting the elusive ‘silver bullet’, or the ‘quick-fix’. Kligler broke free from a colonial attitude a Century ago, which then existed around the world, opting instead to seek the co-operation of everyone, and by treating everyone with dignity and respect, thereby successfully embarking on malaria elimination. If you wish it, it is no dream.

## References

[1] World Health Organization: (2012). Handbook for Integrated Vector Management..

[2] Public Health in Palestine: (1926). The British Medical Journal.

[3] Austen EE: (1919). Anti-mosquito measures in Palestine during the campaigns of 1917-1918.. Trans. R. Soc. Trop. Med. Hyg..

[4] Kligler IJ (1925). The fight against malaria.. The Menorah Journal.

[5] Alexander A, Dunkel F (2017). Local Malaria Elimination: A Historical Perspective from Palestine 100 Years Ago Informs the Current Way Forward in Sub-Saharan Africa,. Am. Entomol..

[6] Levy AJ (1933). The Role of Health Education in the Control of Malaria,. J. Egypt. Med. Assoc..

[7] Kligler IJ (1930). The epidemiology and control of malaria in Palestine..

[8] Russell FF (1925). Rockefeller Foundation Archive Centre, RFA,.

[9] Palestine Antimalarial Advisory Commission: (1925). Proceedings of the 11th Meeting of the Palestine Antimalarial Advisory Commission, 19 May 1925. Abstract in:. Trop. Dis. Bull..

[10] League of Nations: (1925). Malaria Commission. Reports on the Tour of Investigation in Palestine in 1925..

[11] League of Nations: (1927). Report to the Health Committee by Dr. A. Lutrario on the work of the Malaria Commission..

[12] Palestine Department of Health: (1941). A review of the control of malaria in Palestine (1918–1941)..

[13] Palestine Department of Health: (1928). Annual Report for the year 1928..

[14] Report of the Commission on the Palestine Disturbances of August 1929: (1930). Presented by the Secretary of State for the Colonies to Parliament by Command of His Majesty March 1930..

[15] Palestine Department of Health: (1923). Annual Report for the year 1929..

[16] Palestine Royal Commission: (1937). Report Presented by the Secretary of State for the Colonies to Parliament by Command of His Majesty, July 1937..

[17] Dunkel F, Coulibaly K, Montagne C, Luong K (2013). Sustainable integrated malaria management by villagers in collaboration with a transformed classroom using the holistic process: Sanambele, Mali, and Montana State University, USA.. Am. Entomol..

